# A Sulfated Polysaccharide from *Gelidium crinale* Suppresses Oxidative Stress and Epithelial–Mesenchymal Transition in Cultured Retinal Pigment Epithelial Cells

**DOI:** 10.3390/md23100381

**Published:** 2025-09-26

**Authors:** Yurong Fang, Haiyan Zheng, Yizhu Chen, Bomi Ryu, Zhong-Ji Qian

**Affiliations:** 1 School of Chemistry and Environment, Guangdong Ocean University, Zhanjiang 524088, China; yrfang10@163.com (Y.F.); hhyyzzheng@163.com (H.Z.); m13502212988@163.com (Y.C.); 2 Major of Food Science and Nutrition, Pukyong National University, Busan 48513, Republic of Korea

**Keywords:** *Gelidium crinale*, sulfated polysaccharide, epithelial-mesenchymal transition, antioxidant

## Abstract

Age-related macular degeneration (AMD) progresses to vision-threatening dry and wet forms, with no effective dry AMD treatments available. The sulfated polysaccharide (GNP, 25.8 kDa) derived from *Gelidium crinale* exhibits diverse biological activities and represents a potential source of novel therapeutic agents. This study employed a hydrogen peroxide (H_2_O_2_)-induced oxidative stress and epithelial–mesenchymal transition (EMT) model in retinal pigment epithelial (RPE) cells to investigate GNP’s protective mechanisms against both oxidative damage and EMT. The results demonstrated that GNP effectively suppressed oxidative stress, with the 600 μg/mL dose significantly inhibiting excessive reactive oxygen species (ROS) generation to levels comparable to untreated controls. Concurrently, at concentrations of 200–600 μg/mL, GNP inhibited NF-κB signaling and increased the Bax/Bcl-2 ratio, effectively counteracting H_2_O_2_-induced oxidative damage and cell apoptosis. Furthermore, in H_2_O_2_-treated ARPE-19 cells, 600 μg/mL GNP significantly reduced the secretion of N-cadherin (N-cad), Vimentin (Vim), and α-smooth muscle actin (α-SMA), while increasing E-cadherin (E-cad) expression, consequently inhibiting cell migration. Mechanistically, GNP activated the Nrf2/HO-1 pathway, thereby mitigating oxidative stress. These findings suggest that GNP may serve as a potential therapeutic agent for dry AMD.

## 1. Introduction

Age-related macular degeneration (AMD) is a degenerative retinal disease [1] and the leading cause of blindness in individuals over 60 years old in developed countries. The macula, the central functional zone of the retina, is located at the posterior pole and measures approximately 5 mm in diameter. Its central depression, the fovea (only 0.1 mm thick), serves as the focal point for light passing through the ocular refractive system. AMD damages the macula, leading to central vision loss [2]. Insoluble aggregate deposits between Bruch’s membrane and the retinal pigment epithelium (RPE), termed drusen, are the hallmark clinical feature of AMD. Drusen primarily consist of lipids, including cholesterol, calcium phosphate, and various proteins [3]. The type, size, and quantity of drusen are predictive factors for AMD progression [4]. AMD progresses through early to late stages. Early-stage AMD is characterized by intermediate drusen (63–125 μM) without pigment abnormalities, while intermediate-stage AMD presents with large drusen (>125 μM) or intermediate drusen accompanied by pigment abnormalities. Late-stage AMD is classified into two forms: neovascular (wet) and atrophic (dry). Neovascular AMD, accounting for 10–20% of late-stage cases [5], is driven by vascular endothelial growth factor (VEGF) overexpression, which stimulates pathological angiogenesis, resulting in vascular leakage, hemorrhage, or scarring and causing acute vision loss. Dry AMD, the more prevalent form (80–90% of late-stage cases), is characterized by RPE cell death, leading to disrupted photoreceptor nutrition, subsequent atrophy, and irreversible cell loss in critical retinal regions. This manifests as geographic atrophy (GA) and progressive central vision decline [2]. The transition from early to late-stage AMD exhibits a number of shared characteristics, a defective wound-healing response resulting from underlying oxidative stress, degeneration, and chronic inflammation. Subretinal fibrosis is a characteristic of the end-stage of AMD and leads to permanent vision loss. Wound healing is triggered when injured tissue initiates the recruitment and activation of inflammatory cells and fibroblasts. In AMD, fibrosis arises from a dysregulated and excessive wound-healing process [6]. The current standard treatment for wet AMD involves VEGF inhibition. Anti-VEGF agents prevent neovascular growth and mitigate further macular damage and vision loss. However, while these therapies improve visual outcomes, they cannot repair existing damage [7]. Meanwhile, dry AMD affects a substantial patient population, often culminating in legal blindness (visual acuity ≤ 0.1), severely impairing daily life. Notably, some dry AMD cases may progress to wet AMD, underscoring the importance of early intervention to mitigate dual risks. Despite this need, treatment options for dry AMD remain extremely limited, offering only delay but no cure. The complement system serves as a critical effector of innate immunity, playing key roles in cellular homeostasis, tissue development and repair, reproductive processes, and crosstalk with other physiological systems. Activation can occur through three distinct pathways—classical (CP), alternative (AP), and lectin (LP)—each involving unique recognition molecules and initiating serine proteases. These pathways converge at a common terminal sequence, culminating in the generation of the potent anaphylatoxin C5a and the membrane attack complex (MAC, C5b-9). The MAC forms transmembrane pores that can either provoke “sub-lytic” cellular activation or cause direct lysis of target cells [8]. In particular, genomic and proteogenomic polymorphisms that influence the alternative complement pathway are strongly associated with the development and progression of AMD [9]. Complement 3 (C3), complement factor F, complement factor H (CFH), and MAC were identified in both Drusen and AMD lesions. Additionally, elevated levels of C3, C3d, Bb, and C5a were found in the plasma of AMD patients. These findings, particularly the dysregulation of the alternative pathway leading to increased C3 turnover, suggest a prominent pathogenic role in AMD [10]. In February 2023, the C3-targeting therapy pegcetacoplan (SYFOVRE) received U.S. Food and Drug Administration (FDA) approval as the first treatment for GA. In August 2023, avacincaptad pegol (Izervay) received approval for the treatment of GA, which targets complement component C5, is the second to be approved for this condition. Both treatments involve intravitreal injections (IVI) [11]. Although early clinical trials showed promise, both drugs reported serious ocular adverse events (AEs), including new-onset neovascular AMD, intraocular inflammation, ischemic optic neuropathy, and elevated intraocular pressure [12]. Dietary supplementation, particularly formulations including lutein, zeaxanthin, and beta-carotene, offers a low-risk strategy for managing early and intermediate AMD [13]. Currently, the findings from the AREDS and AREDS2 trials highlight the potential of nutritional interventions to slow GA progression, particularly toward the central macula [14]. They provide modest slowing of progression in early-stage patients but no benefit for established atrophy [15]. Given the complex pathophysiology of dry AMD—involving oxidative stress, inflammation, metabolic dysregulation, and multifactorial pathways—identifying preventive and therapeutic agents for dry AMD is of critical importance.

Age-related macular degeneration (AMD) is a complex, multifactorial disorder whose onset and progression may be triggered by diverse factors including aging, genetic predisposition, smoking, oxidative damage, and inflammation. Among these, oxidative stress represents a pivotal pathogenic mechanism in AMD [16]. The RPE consists of highly specialized epithelial cells that exhibit unique structural and functional polarity. Their apical surfaces form intimate connections with photoreceptor outer segments (POS), while their basal surfaces adhere to Bruch’s membrane. These polarized cells play essential roles in maintaining daily photoreceptor renewal and supporting the nutritional and functional integrity of the choriocapillaris [17]. Through phagocytosis of shed POS, RPE cells ensure continuous photoreceptor renewal. However, the exceptionally high metabolic rate of photoreceptors in the retina generates substantial amounts of reactive oxygen species (ROS) [18]. Under physiological conditions, ROS generation constitutes an integral component of normal cellular metabolism and redox signaling, serving to maintain energy homeostasis and cellular functions. The RPE is normally equipped with abundant antioxidant defenses that effectively scavenge oxygen free radicals to preserve redox balance. However, in AMD, RPE cells demonstrate increased levels of apoptosis, impaired autophagy, and incomplete POS digestion, collectively exacerbating oxidative stress burden [19]. Chronic oxidative stress compromises the phagocytic and autophagic functions of RPE cells, leading to protein aggregation and activation of inflammatory vesicles. This pathological cascade promotes the generation of free radicals and oxidized toxic byproducts within RPE cells, ultimately driving AMD initiation and progression. Notably, oxidative stress can induce epithelial–mesenchymal transition (EMT) in RPE cells. During EMT, cells undergo phenotypic transformation characterized by loss of apical–basal polarity, disruption of cell–cell adhesions, and acquisition of mesenchymal traits including migratory capacity. This process has been implicated in retinal cellular alterations and damage, thereby contributing to age-related pathologies such as AMD. While, type 1 EMT has a role during development and type 3 EMT occurs in most cancers, type 2 EMT is associated with wound healing and tissue regeneration. Type 2 EMT represents a physiological process involved in tissue regeneration and wound healing; however, its aberrant activation can exacerbate pathological fibrosis [15]. Although described as “degeneration”, a closer examination of these “degeneration” cells reveals that some of them do not die but may transform into mesenchymal cells in order to survive in the harsh microenvironment during disease progression [20]. Since EMT is a reversible pathophysiological process [21], these cells represent a breakthrough target for novel therapies aimed at reversing dry AMD. 

In recent decades, the vast marine environment has yielded numerous bioactive compounds with high efficacy and low toxicity [22]. Among these, seaweed—as one of the most important marine plants—has been extensively studied for its diverse bioactive components [15,23]. Red algae (Rhodophyta) represent the largest category of marine macroalgae in China [24]. These algae are particularly rich in polysaccharides, including sulfated galactans, which are among the most abundant non-mammalian sulfated polysaccharides found in nature. Marine-derived sulfated polysaccharides exhibit multiple biological and physiological activities, including anticoagulant, antiviral, antioxidant, anti-inflammatory, and antitumor properties. *Gelidium crinale*, an economically important and traditionally edible red alga belonging to the Gelidiaceae family [25], has attracted research attention. Previous studies have identified GNP (a sulfated polysaccharide isolated from *Gelidium crinale*) with a relative molecular mass of 25.8 kDa. Its monosaccharide composition is predominantly galactose (63.05%), and it contains abundant sulfate ester groups, consistent with the fundamental characteristics of red algal polysaccharides. Preliminary research has demonstrated that GNP possesses significant antioxidant and anti-inflammatory activities [26]. The antioxidant activity of polysaccharides may be attributed to specific structural traits, particularly higher proportions of glucose, galactose, and uronic acid, as demonstrated in previous studies [27,28]. Notably, the sulfate groups in algal polysaccharides are closely associated with their biological activities. Wang et al. found that after ultrasonic treatment reduced the molecular weight of yellow tea polysaccharides, this in vitro antioxidant activity significantly increased. This effect was attributed to an increase in hydrogen bonds following polysaccharide degradation [29]. Five kinds of algal polysaccharides, *Ulva pertusa*, *Laminaria japonica*, *Grateloupia filicina*, *Bryopsis plumosa*, and *Porphyra haitanensis* have been extracted and determined by in vitro antioxidant activities [30]. The results indicated that all samples exhibited antioxidant activity and strong free radical scavenging ability, which remained stable even at high temperatures. In the reducing power assay, *Laminaria japonica* polysaccharide (LJP) demonstrated the strongest reducing capacity, likely due to its higher sulfate content and lower hydroxyl group content, thereby providing fewer hydrogen atoms [31]. Given that dry age-related macular degeneration (AMD) pathogenesis is closely associated with oxidative stress mechanisms, and considering that the antioxidant activity of natural polysaccharides correlates with monosaccharide composition and relative molecular weight, while the bioactivity of sulfated polysaccharides strongly correlates with their sulfate group content and position, it is suggested that GNP—characterized by a high galactose proportion in its monosaccharide profile, relatively low molecular weight, and its high sulfate content—may demonstrate strong antioxidant activity, showing substantial potential for dry AMD prevention and treatment.

ARPE-19 cells represent one of the most widely used cellular models in vision research and serve as a standard system for AMD studies. Hydrogen peroxide (H_2_O_2_) can impair cellular functions by enhancing oxidative stress in RPE cells, leading to protein damage and aggregation-key features of AMD pathogenesis [32]. In this study, we employed an H_2_O_2_-induced oxidative stress model in ARPE-19 retinal pigment epithelial cells to preliminarily investigate the effects of GNP on oxidative stress, inflammatory responses, apoptosis, and epithelial–mesenchymal transition (EMT).

## 2. Results

### 2.1. Effect of GNP on H_2_O_2_-Induced ARPE-19 Viability

**Figure 1:**
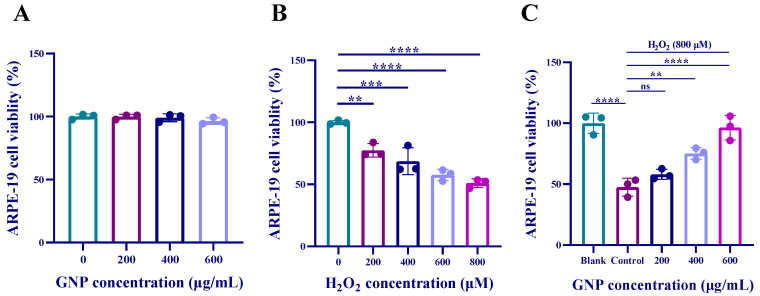
Effect of GNP on the viability of ARPE-19 cells. (**A**) The effect of GNP on the activity of ARPE-19 cells; (**B**) The effect of H_2_O_2_ stimulation on the activity of ARPE-19 cells; (**C**) The protective effect of GNP preconditioning on ARPE-19 cells under H_2_O_2_ stimulation. Data are shown as mean ± SD (*n* = 3). *p* < 0.01 (**), *p* < 0.001 (***), *p* < 0.0001 (****) compared with H_2_O_2_ alone treated control group.

The results showed that there was no significant change in the viability of ARPE-19 cells, indicating that GNP treatment at concentrations up to 600 µg/mL had no toxic effects (Figure 1A). Therefore, all subsequent experiments used GNP concentrations of 200, 400, and 600 µg/mL. As shown in Figure 1B, hydrogen peroxide reduced cell viability in a dose-dependent manner. When cells were exposed to 800 µM hydrogen peroxide, the survival rate was approximately 50%. As demonstrated in Figure 1C, GNP treatment significantly improved the viability of ARPE-19 cells after exposure to 800 µM hydrogen peroxide. These results suggest that GNP (200, 400, and 600 µg/mL) effectively prevent hydrogen peroxide-induced damage to ARPE-19 cells.

### 2.2. Effect of GNP on the Migration Ability in H_2_O_2_-Induced ARPE-19

**Figure 2:**
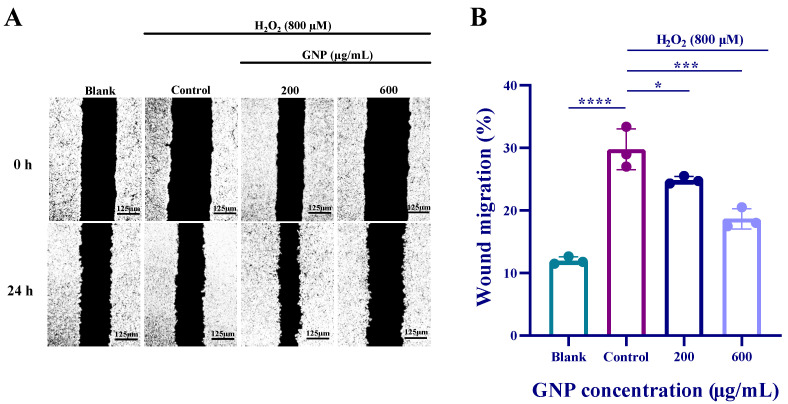
Effect of GNP on the migratory capacity of ARPE-19 cells. (**A**) Representative images of the effect of GNP on cell migration; (**B**) The cell scratch healing rate of GNP. Data are shown as mean ± SD (*n* = 3). *p* < 0.05 (*), *p* < 0.001 (***), *p* < 0.0001 (****) compared with H_2_O_2_ alone treated control group.

Cell scratch assay was used to observe the effect of GNP on ARPE-19 cells migration. Compared with the blank group, the addition of H_2_O_2_ caused ARPE-19 cells to rapidly migrate to the “wound” area. However, when GNP were added, the migratory ability of ARPE-19 cells was significantly reduced. At a concentration of 600 μg/mL, GNP treatment demonstrated a significant inhibition of ARPE-19 cells migration (Figure 2). It indicates that GNP inhibits the migration ability of ARPE-19 cells in a dose-dependent manner.

### 2.3. Effect of GNP on ROS Production in H_2_O_2_-Induced ARPE-19

**Figure 3:**
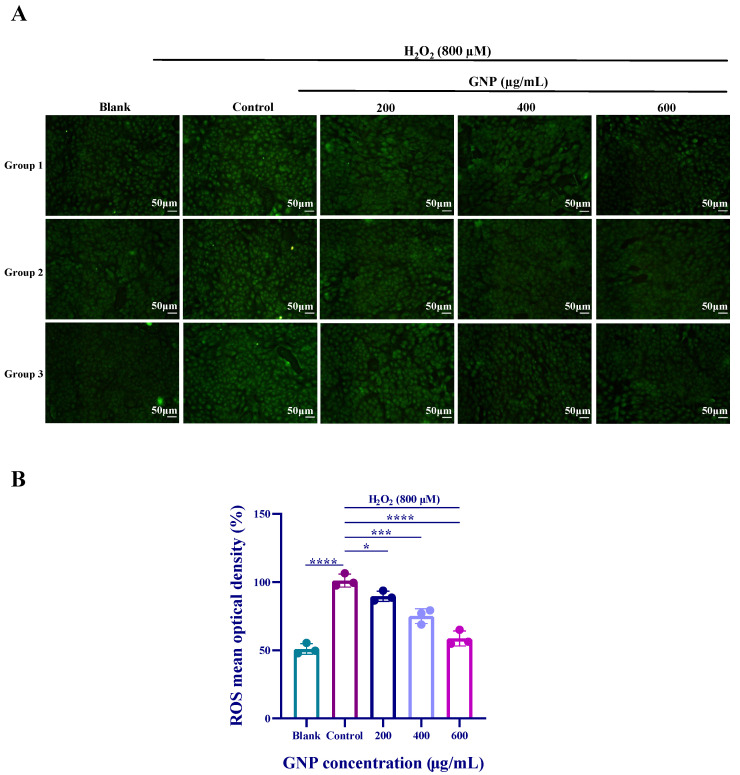
Effects of H_2_O_2_, GNP on intracellular ROS content. (**A**) Cellular ROS fluorescence intensity; (**B**) Cellular fluorescence intensity values. Data are shown as mean ± SD (*n* = 3). *p* < 0.05 (*), *p* < 0.001 (***), *p* < 0.0001 (****) compared with H_2_O_2_ alone treated control group.

According to the fluorescence measurement results, it can be observed that the fluorescence intensity of the H_2_O_2_ stimulated group was significantly higher than that of the control group (Figure 3). After treatment with different concentrations of GNP for 24 h, ROS levels exhibited a dose-dependent decrease. The results indicate that GNP exerted a protective effect on H_2_O_2_-induced ARPE-19 cells by inhibiting ROS.

### 2.4. Effect of GNP on NF-κB Pathway in H_2_O_2_-Induced ARPE-19

**Figure 4:**
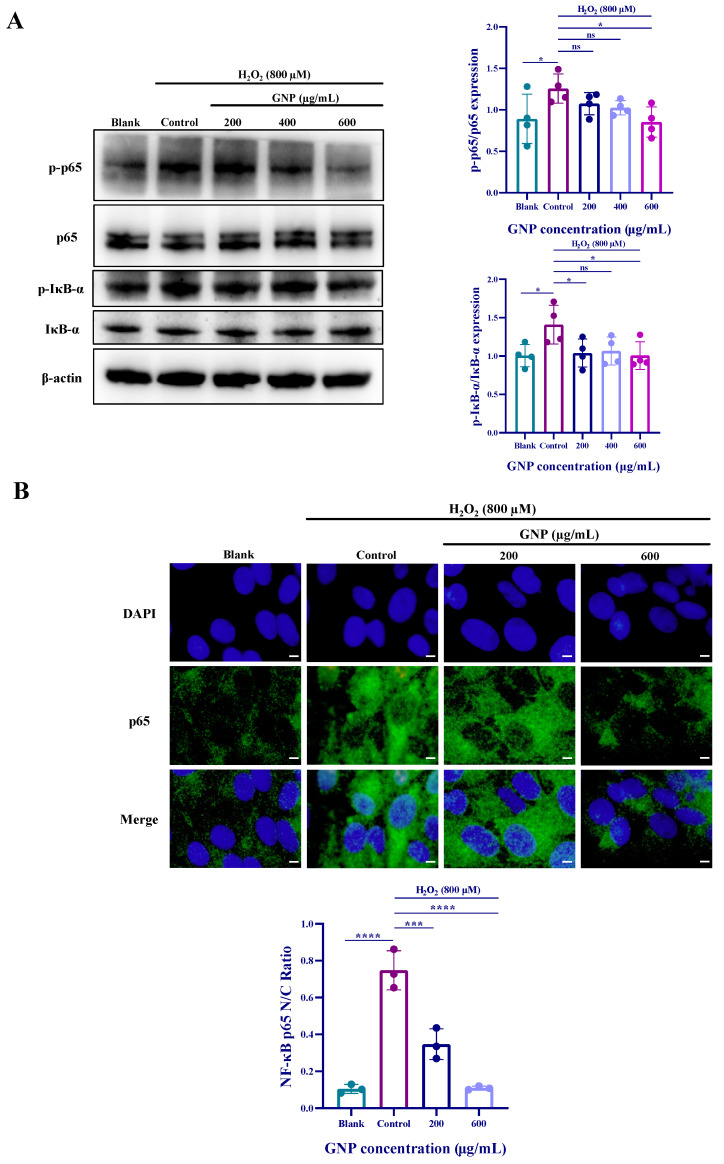
Effect of GNP on H_2_O_2_-induced NF-κB signaling pathway expression in ARPE-19 cells. (**A**) The protein expression level of NF-κB signaling pathway in cell lysates of ARPE-19 cells was measured by Western blot analysis. β-actin was used as an internal control. (**B**) Immunofluorescence strain for p65 protein in H_2_O_2_-induced ARPE-19 cells. DAPI staining for nucleus, Dylight 488 staining for p65 protein, merging image showed the change in p65 expression. (**C**) Analysis of nucleus/cytoplasmic ratios in p65 immunofluorescence images. Data are shown as mean ± SD (*n* = 3–4). *p* < 0.05 (*), *p* < 0.001 (***), *p* < 0.0001 (****) compared with H_2_O_2_ alone treated control group.

Compared to untreated control groups, H_2_O_2_ stimulation of ARPE-19 cells led to increased expression of p-p65 and p-iκB-α. However, treatment with GNP at concentrations of 200, 400, and 600 μg/mL resulted in reduced phosphorylation levels of p65 and iκB-α (Figure 4A). To further validate the effect of GNP on the NF-κB signaling pathway, immunofluorescence experiments were conducted (Figure 4B). The results demonstrated that H_2_O_2_ stimulation caused translocation of p65 into the nucleus in ARPE-19 cells. However, after 24 h of GNP treatment, nuclear p65 levels decreased in a dose-dependent manner. These findings indicate that GNP exerts an inhibitory effect on the NF-κB pathway.

### 2.5. Effects of GNP on the Expression of Nrf2 and HO-1 Proteins in H_2_O_2_-Induced ARPE-19

**Figure 5:**
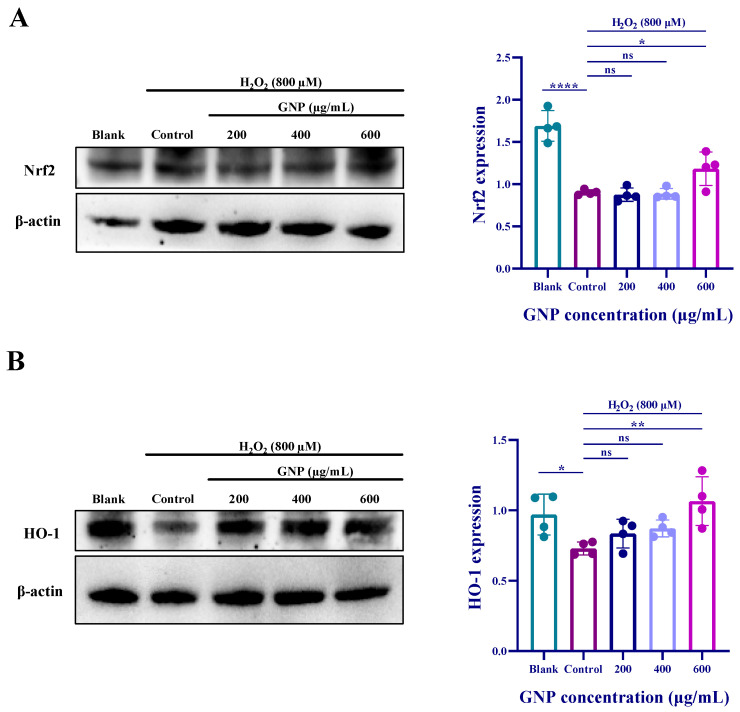
Effect of GNP on H_2_O_2_-induced expression of antioxidant-related proteins in ARPE-19 cells. (**A**) The protein expression level of Nrf2 in cell lysates of ARPE-19 cells was measured by Western blot analysis. β-actin was used as an internal control. (**B**) The protein expression level of HO-1 in cell lysates of ARPE-19 cells was measured by Western blot analysis. β-actin was used as an internal control. Data are shown as mean ± SD (*n* = 4). *p* < 0.05 (*), *p* < 0.01 (**), *p* < 0.0001 (****) compared with H_2_O_2_ alone treated control group.

The mechanism of the antioxidant pathway was investigated using Western blot experiments. Results (Figure 5) demonstrate that H_2_O_2_ treatment downregulated the expression of Nrf2 protein, and HO-1 protein expression exhibited a similar trend to Nrf2. Pretreatment with GNP increased the expression of both Nrf2 and HO-1 proteins, with the most significant effect observed at 600 μg/mL GNP. These findings suggest that GNP may exert its antioxidant effects by regulating the Nrf2/HO-1 pathway, thereby alleviating oxidative stress in ARPE-19 cells.

### 2.6. Effect of GNP on Apoptosis of H_2_O_2_-Induced ARPE-19

**Figure 6:**
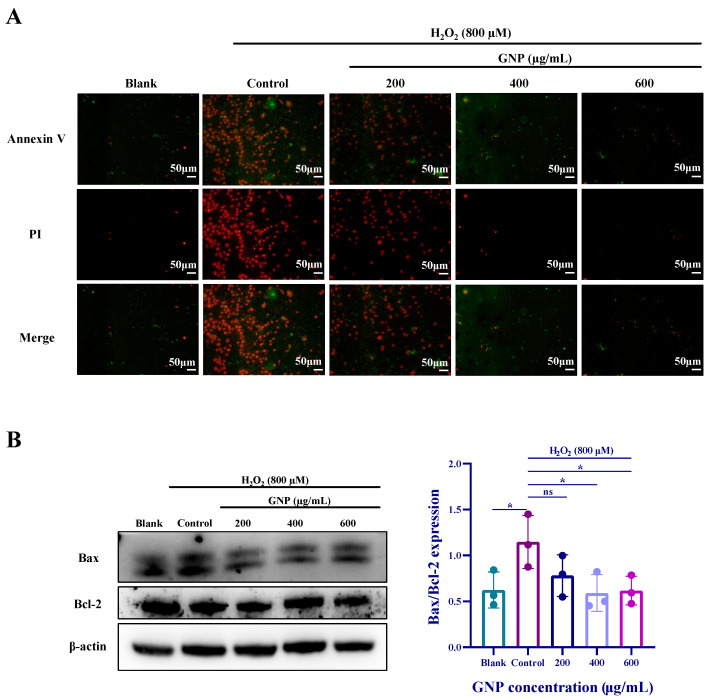
Effect of GNP on apoptosis of H_2_O_2_-induced ARPE-19 cells. (**A**) The protective effecuts of GNP on H_2_O_2_-induced apoptosis were detected by Annexin V-FITC/PI staining. (Green: early apoptosis; Red: late apoptosis). (**B**) The protein expression level of Bax/Bcl-2 in cell lysates of ARPE-19 cells was measured by Western blot analysis. β-actin was used as an internal control. Data are shown as mean ± SD (*n* = 3). *p* < 0.05 (*) compared with H_2_O_2_ alone treated control group.

Cell damage is often accompanied by apoptosis, and Annexin V is one of the sensitive indicators for detecting early apoptosis, labeled with green fluorescence (FITC). Propidium Iodide (PI), a nucleic acid dye, stains cell nuclei during the mid-to-late stages of apoptosis and necrotic cells. The staining results can be detected using fluorescence microscopy or flow cytometry, as shown in Figure 6A. Compared to the blank group, the H_2_O_2_-treated group exhibited significantly increased red and green fluorescence, indicating severe apoptosis progressing into the late stage. After treatment with GNP, fluorescence intensity decreased, with the most notable reduction observed in the GNP-treated group at a concentration of 600 μg/mL. Western blot analysis of apoptosis-related protein expression (Figure 6B) showed that compared to the blank group, the control group exhibited reduced Bcl-2 protein expression and increased Bax protein expression. Compared to the control group, ARPE-19 cells treated with GNP displayed elevated Bcl-2 expression and decreased Bax expression, with these changes being dose-dependent.

### 2.7. Effect of GNP on H_2_O_2_-Induced EMT Biomarkers in ARPE-19 Cells

**Figure 7:**
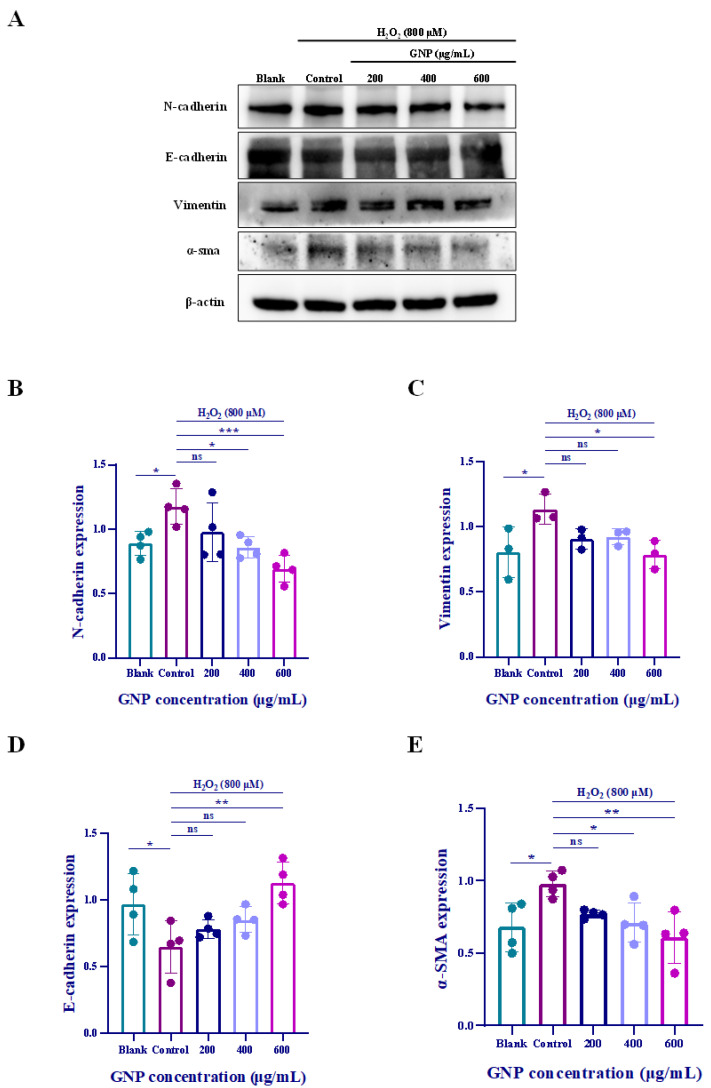

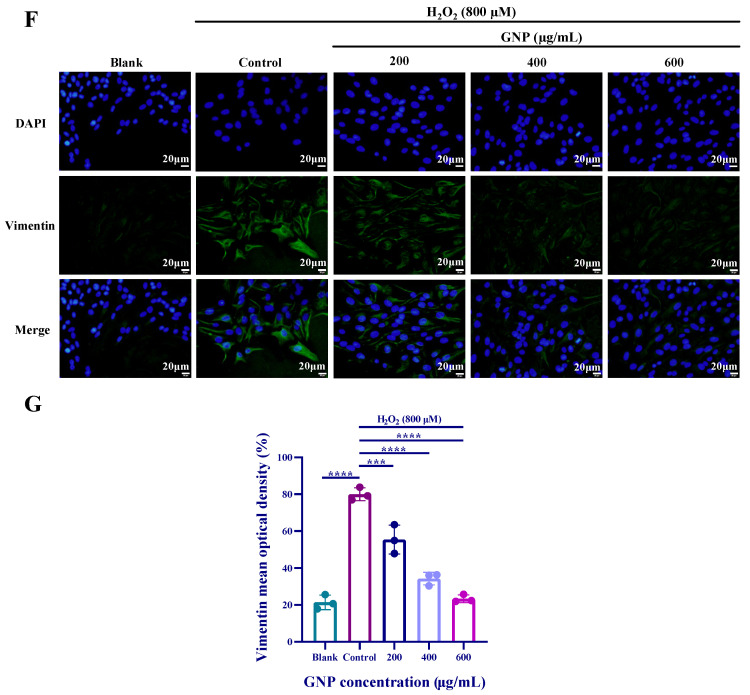
Effect of GNP on EMT biomarker in H_2_O_2_-induced ARPE-19 cells. (**A**–**E**) N-cadherin, E-cadherin, Vimentin, α-SMA protein expression in cell lysates of H_2_O_2_-induced ARPE-19 cells were measured by Western blot analysis. β-actin was used as an internal control. (**F**) Immunofluorescence strain for Vimentin protein in H_2_O_2_-induced ARPE-19 cells. DAPI staining for nucleus, Dylight 488 staining for Vimentin protein, merging image showed the change in Vimentin expression. (**G**) Analysis of mean fluorescence intensity in Vimentin immunofluorescence images. Data were shown as mean ± SD (*n* = 3–4). *p* < 0.05 (*), *p* < 0.01 (**), *p* < 0.001 (***), *p* < 0.0001 (****) compared with H_2_O_2_ alone treated control group.

To determine the potential impact of GNP on epithelial–mesenchymal transition (EMT), we employed Western blot and immunofluorescence assays to detect the expression levels of EMT markers in ARPE-19 cells. Western blot analysis revealed that H_2_O_2_-treated ARPE-19 cells exhibited significant upregulation of α-SMA, N-cadherin, and Vimentin, along with a marked downregulation of E-cadherin (Figure 7A–E). Similarly, immunofluorescence assays demonstrated increased Vimentin expression (Figure 7F). These findings indicate that ARPE-19 cells undergo an epithelial phenotype shift under oxidative stress. However, the expression of these markers was reversed in ARPE-19 cells pretreated with GNP. Collectively, these results demonstrate that GNP suppresses H_2_O_2_-induced EMT in ARPE-19 cells.

## 3. Discussion

More than 70% of the Earth’s surface is covered by oceans, which host marine ecosystems of exceptionally high biodiversity [33]. Among marine organisms, seaweeds represent one of the most important plant resources and are recognized as a significant source of bioactive compounds [34]. Red algae (Rhodophyta), as a major category of seaweeds, serve as an important source of biologically active marine polysaccharides [35]. *Gelidium crinale* is an edible seaweed rich in sulfated polysaccharides. Previous studies have demonstrated that GNP, a sulfated polysaccharide extracted from edible *Gelidium crinale*, exhibits multiple pharmacological activities including anti-inflammatory, antioxidant, antitumor, and anti-atherosclerotic effects [36]. Our early studies have shown that GNP is a mixture of different polysaccharides mainly containing galactose (65.05%), xylose (11.55%), fucose (11.19%), glucose (6.73%), glucuronic acid (5.54%), rhamnose (0.79%), ribose (0.47%), amino galactose (0.43%) and arabinose (0.26%) [15,34]. This may underlie the antioxidant activity of GNP [25]. Furthermore, the configuration of glycosidic linkages significantly influences polysaccharide bioactivity; in general, β-configured polysaccharides exhibit superior biological activity. Fourier transform infrared (FTIR) spectroscopy confirmed the presence of β-linkages in GNP, which may further contribute to its enhanced efficacy [37]. Marine algal polysaccharides exhibit considerable diversity in their chemical composition, influenced by variations in algal species, geographic origin, and harvest season—factors that are critically linked to their biological activities. The composition of the monosaccharide affects the antioxidant activity of the marine polysaccharides. For instance, polysaccharide P2 from *Pavlova viridis* [38] and polysaccharide UFP2 from *Ulva fasciata* [39] possess similar molecular weights (55.0 and 54.7 kDa, respectively) and sulfate content (17.80% and 16.28%, respectively), yet differ significantly in their monosaccharide composition. P2 is primarily composed of glucose, rhamnose, D-fructose, and mannose, whereas UFP2 consists mainly of rhamnose, glucosamine, and xylose. Notably, P2 exhibits markedly stronger free radical scavenging activity, demonstrating 96% scavenging of DPPH radicals compared to 20–25% by UFP2, and 98% scavenging of hydroxyl radicals versus 40–45% for UFP2 [40].

ROS play a pivotal role in the pathogenesis of AMD, particularly in the dry (atrophic) form, where oxidative damage directly leads to RPE dysfunction and cell death. ROS are byproducts of mitochondrial oxidative phosphorylation and are normally cleared by antioxidant systems. However, with aging or increased oxidative stress, ROS production may exceed clearance capacity, resulting in oxidative damage. Due to the proximity of mitochondrial DNA (mtDNA) to ROS generation sites and its lack of protective histones, mtDNA is more vulnerable to ROS-induced damage than nuclear DNA. In AMD, mtDNA damage and mutations accumulate progressively. Under pathological aging and oxidative stress conditions, the efficiency of mtDNA repair mechanisms declines, further exacerbating the accumulation of mtDNA damage, including fragmentation, mutations, and increased single nucleotide polymorphisms (SNPs). These mtDNA defects ultimately impair mitochondrial function, disrupting energy supply and cellular homeostasis in RPE cells. RPE dysfunction is a central mechanism in AMD pathogenesis, eventually leading to retinal degeneration and vision loss [41]. ARPE-19 is a spontaneously immortalized cell line of human RPE that is used widely to draw inferences about the behavior of adult human RPE (ahRPE). Hierarchic clustering analysis demonstrated that the gene expression profile of ahRPE and ARPE19 samples cluster into two distinct groups with no discernable overlap. The expression of 5634 ± 65 gene probes (out of 12,600 on microarray Human U95Av2 chip) was detected in ARPE-19 cells compared to 5580 ± 84 genes in ahRPE cells from four human donor eyes. Thirty-five genes are expressed exclusively in ahRPE and nine genes exclusively in ARPE-19 cells. Fifty additional genes have a threefold increase and 40 genes have a threefold decrease in expression level in ahRPE compared to ARPE-19. This suggests that there are significant differences in gene expression between ARPE-19 cells and human RPE cells [42]. Transcriptomic validation demonstrated that both ahRPE cells and induced pluripotent stem cell-derived RPE (iPSC-RPE) cells exhibit comparable gene expression profiles, showing significantly higher levels than ARPE-19 cells. Notably, ARPE-19 cells exhibit lower transepithelial electrical resistance (TEER) compared to both ahRPE cells and iPSC-RPE cells, indicating distinct differences in gene expression patterns and barrier function between ARPE-19 cells and ahRPE cells [43]. Therefore, further validation in animal studies and drug research is crucial.

The Nrf2/HO-1 signaling pathway has been previously demonstrated to mitigate oxidative stress. Under oxidative stress, when cells are attacked by ROS, Nrf2 rapidly translocates to the nucleus. Phosphorylated Nrf2 further activates the expression of heme oxygenase-1 (HO-1), which catalyzes heme degradation to produce biliverdin, carbon monoxide (CO), and free iron (Fe^2+^) [44]. Bilirubin is oxidized to biliverdin, which can then be reduced back to bilirubin by biliverdin reductase in vivo, forming a “bilirubin-biliverdin cycle”. Biliverdin scavenges hydroxyl radicals, singlet oxygen, and superoxide anions, thereby preventing excessive protein oxidation and exerting potent antioxidant, anti-inflammatory, and anti-apoptotic effects [45]. Previous studies have shown that GNP reduces ROS levels and attenuates oxidative damage in ox-LDL-induced human vascular endothelial cell atherosclerosis [26]. He and others demonstrated that H_2_O_2_ stimulation upregulates nuclear Nrf2 expression, thereby increasing HO-1 levels, indicating that Nrf2/HO-1 pathway activation protects against oxidative stress [46]. In this study, GNP downregulated H_2_O_2_-induced ROS levels while upregulating Nrf2 and HO-1 expression, suggesting that GNP exerts antioxidative effects in AMD-related RPE cells.

Excessive oxidative stress (OS) disrupts cellular processes, thereby inducing inflammation and apoptosis. ROS activate the Nuclear Factor-κB (NF-κB) pathway, which plays a crucial role in inflammatory responses within the immune system. The Nrf2/HO-1 pathway inhibits the NF-κB pathway, while NF-κB also exerts reciprocal regulation on Nrf2/HO-1. Under oxidative stress, both pathways engage in coordinated adaptation, ultimately establishing a dynamic equilibrium. The activation of NF-κB pathway can also trigger apoptosis. Under normal physiological conditions, the NF-κB signaling pathway remains in an inactive state in the cytoplasm by forming a complex with the inhibitory protein IκB as a p65-p50 heterodimer. Upon appropriate stimulation, activated NF-κB p65 translocates to the nucleus [47]. In this study, immunofluorescence results demonstrated that GNP suppressed the nuclear translocation of p65 in ARPE-19 cells. Western blot analysis further revealed that GNP reduced the phosphorylation levels of both p65 and IκB-α. Apoptosis is not a passive process but rather an active one involving the activation, expression, and regulation of a series of genes [48]. The Bcl-2 family serves as a key regulator of apoptosis, comprising pro-apoptotic members (e.g., Bax) and anti-apoptotic members (e.g., Bcl-2). The Bcl-2/Bax ratio is considered a critical indicator of anti-apoptotic capacity, with a higher ratio signifying stronger anti-apoptotic potential. Western blot results showed that H_2_O_2_ treatment decreased the Bcl-2/Bax ratio, whereas GNP pretreatment upregulated Bcl-2 expression, increased the Bcl-2/Bax ratio, and downregulated Bax expression, collectively inhibiting apoptosis.

EMT is a process by which epithelial cells undergo transformation into mesenchymal phenotypes, strongly associated with OS. During EMT, epithelial cells lose their apical-basal polarity and cell–cell adhesion properties while acquiring mesenchymal characteristics, including enhanced motility, invasiveness and resistance to apoptosis. This process is characterized by upregulation of mesenchymal markers such as vimentin (Vim), N-cadherin (N-cad) and α-smooth muscle actin (α-SMA), accompanied by downregulation of epithelial markers E-cadherin (E-cad), ultimately leading to increased invasiveness, metastatic dissemination, cancer stemness, and chemoresistance [49]. EMT plays a significant role in various ocular degenerative diseases, including AMD. In this study, the results showed that GNP promoted E-cadherin levels and inhibited the expression of N-cadherin, Vimentin, α-SMA, and controlled the migration and proliferation of ARPE-19 cells (Figure 7). E-cadherin serves as a key adhesion structure between epithelial cells, providing strong and stable intercellular connections. In contrast, N-cadherin, which is predominantly expressed in mesenchymal cells, confers weaker and more dynamic adhesion capabilities. Meanwhile, vimentin offers mechanical protection to migrating cancer cells against stresses encountered during migration or spatial confinement, and is also widely recognized as a critical marker of EMT [36]. Furthermore, wound healing assays demonstrated that GNP inhibited H_2_O_2_-induced cell migration (Figure 2), indicating that GNP effectively suppresses EMT triggered by oxidative stress.

Currently, research on polysaccharides for the treatment of AMD largely remains at the stage of in vitro cell experiments or animal models. The therapeutic effects of polysaccharides on dry AMD lack clear and precise mechanistic explanations and have not been rigorously validated in clinical trials. Meanwhile, the interaction between polysaccharides and the gut microbiome is widely recognized. As plant polysaccharides have been part of the human diet since the beginning of human evolution, the gut microbiome has evolved diverse strategies to adapt to and metabolize undigested non-starch plant polysaccharides. The gut microbiota produces carbohydrate-active enzymes (CAZymes) that break down polysaccharides into monosaccharides, which can be utilized by microbes as an energy source [50]. When administered orally, polysaccharides must be absorbed through the gastrointestinal tract into the systemic circulation. However, due to their high molecular weight (e.g., dextran, hyaluronic acid), most polysaccharides exhibit extremely low gastrointestinal absorption rates and face even greater challenges in reaching the macular region. Furthermore, polysaccharides are susceptible to degradation by glycosidases in the human body and may be metabolized and inactivated by the gut microbiota after oral intake [51]. It is also important to note that dry AMD is a multifactorial disease involving genetic predisposition, oxidative stress, chronic inflammation, among other mechanisms. A single polysaccharide with limited bioactive properties is unlikely to address all these pathological aspects effectively. Therefore, targeted delivery of polysaccharides to the RPE layer in the macula is essential for their therapeutic efficacy against dry AMD. Future research should focus on elucidating the mechanisms through which polysaccharides modulate key pathological processes in dry AMD via more precise fundamental studies, thereby enabling the development of highly efficient ocular targeted delivery systems leveraging advancements in nanomedicine and materials science. 

Meanwhile, this study has several limitations. First, the experimental model of EMT relied on endogenous pathways triggered by oxidative stress rather than exogenous induction with a canonical activator such as TGF-β [52]. While this approach reflects a physiologically relevant stimulus, the absence of a positive control inducer limits direct comparability with studies using standardized EMT models. Second, the assessment of antioxidant activity, though demonstrating significant effects, lacked benchmarking against well-established antioxidants such as N-acetylcysteine (NAC) [53] or vitamin C [54]. This omission makes it difficult to quantitatively position the potency of GNP relative to known agents. Finally, although we observed a protective effect against cell death, no specific apoptotic experiments such as caspase-3/7 activity or PARP cleavage were conducted. Therefore, the protective effect cannot be explicitly attributed to the anti-apoptotic pathway. Despite these limitations, our findings evaluate the comprehensive bioactivity of GNP and provide preliminary evidence supporting its protective effects against oxidative stress and EMT-like phenotypic changes. Future studies will explicitly address these limitations by incorporating TGF-β as a positive control for EMT induction, using NAC or other antioxidants as quantitative benchmarks, and employing caspase-based assays to confirm anti-apoptotic mechanisms. Such approaches will help precisely delineate the contribution of GNP to specific pathways and facilitate its development as a targeted therapeutic agent.

## 4. Materials and Methods

### 4.1. Materials and Chemicals

GNP was extracted from *Gelidium crinale* of Naozhou Island Sea, Zhanjiang City, Guangdong Province. The process involved treating the samples with 90% ethanol (*w*/*v* = 1:8), followed by drying at 45 °C. Extraction was conducted using 0.1 M HCl (*w*/*v* = 1:8) for 8 h, after which the solution was neutralized and centrifuged. The supernatant was concentrated in a rotary evaporator and precipitated with 80% ethanol. The precipitate was redissolved in distilled water, and proteins were removed using Sevag reagent. After concentration and dialysis, the sample was purified through a Sepharose Cl-6B column (2.5 × 60 cm) with an elution phase of 0.1 mol/L NaCl. The final product was freeze-dried to obtain GNP, achieving a purity of 98.5%. The molecular weight of the GNP was determined to be 25.8 kDa, with galactose being the most abundant monosaccharide component. Additionally, it contained 16.5% sulfate content. ARPE-19 human retinal pigment epithelial cells were purchased from the Cell Bank of the Chinese Academy of Sciences (Shanghai). Fetal bovine serum (FBS), trypsin-EDTA (0.25%), dulbecco’s modified eagle medium (DMEM), and penicillin/streptomycin were from Gibco (New York, NY, USA). Hydrogen peroxide (H_2_O_2_) was purchased from Guangdong Guanghua Science and Technology. Cell Counting Kit-8 (CCK-8) was purchased from ZETA LIFE (San Francisco, CA, USA). DCFH-DA fluorescent probe, RIPA buffer and 4′,6-diamidino-2-phenylindole (DAPI) were purchased from Shanghai Bioyotime Biotechnology (Shanghai, China). The primary antibodies of Bax, Bcl-2, Nrf2, HO-1, p-IκB-α, IκB-α, p-p65, p65, α-SMA, Vimentin, N-cadherin, E-cadherin, β-actin, secondary antibodies were obtained from Santa Cruz Biotechnology Inc. (Dallas, TX, USA) and Cell Signaling Technology.

### 4.2. Cell Culture and Viability Assay

The ARPE-19 cells were cultured in 90% DMEM, 10% FBS, and 1% penicillin/streptomycin complete medium and grown in 37 °C, 5% CO_2_ incubator. After seeding ARPE-19 cells in 96-well plates for 24 h, they were treated with various concentrations of GNP (200, 400, and 600 μg/mL). After 24 h, added 100 μL of 10% CCK-8 solution. After 30 min incubation in darkness at 37 °C, measure absorbance at 450 nm with a microplate reader (BioTek, Winooski, VT, USA). 

### 4.3. Cell Wound Healing Assay 

ARPE-19 cells were plated in 6-well plates and allowed to adhere for 24 h. Following 24 h exposure to varying concentrations of GNP in serum-free medium, a uniform scratch wound was created in the confluent monolayer using a sterile 200 μL pipette tip. Cells were subsequently treated with H_2_O_2_ (800 μM) for 24 h in serum-free medium. The migration of ARPE-19 cells across the wound edge at 0 h and 24 h after the addition of H_2_O_2_ was imaged using a microscope (Olympus, Tokyo, Japan). The formula to calculate healing rate is

Healing Rate (%) = [(A_0_ − A_t_)/A_0_] × 100% (1)

where A_0_ = Initial wound area (at time 0), Aₜ = Remaining wound area at measurement time “t” (e.g., 24 h).

### 4.4. Measurement of Intracellular ROS

ARPE-19 cells were seeded in 24-well plates and treated with GNP at concentrations of 200, 400, and 600 μg/mL. After 24 h of incubation, cells were exposed to 800 μM H_2_O_2_ for 24 h. Subsequently, 10 μM DCFH-DA was added to each well and incubated in the dark at 37 °C for 30 min. Following two washes with PBS, intracellular ROS generation was visualized and imaged using a fluorescence microscope (Olympus, Tokyo, Japan).

### 4.5. Annexin V-FITC/PI Apoptosis Detection

ARPE-19 cells were transferred to 24-well plates and cultured in complete medium for 24 h, followed by treatment according to the method described in Section 2.5. For apoptosis detection, cells were stained using an Annexin V-FITC/PI staining kit by adding 100 μL of binding buffer, 5 μL of Annexin V, and 5 μL of propidium iodide (PI) to each well, followed by incubation at room temperature in the dark for 10 min. Finally, cells were observed under a fluorescence microscope. The Annexin V-FITC fluorescence signal appeared green, while the PI fluorescence signal appeared red.

### 4.6. Immunofluorescence

After treatment with GNP (200, 400, and 600 μg/mL, 24 h), cells were exposed to H_2_O_2_ (800 μM, 24 h) and then removed from the incubator for subsequent experiments. The old medium was aspirated, and cells were fixed with 4% paraformaldehyde (4 °C, 30 min), followed by permeabilization with 0.2% Triton X-100 (4 °C, 10 min). After washing three times with PBS, cells were blocked with 5% bovine serum at room temperature (1 h). Subsequently, cells were incubated overnight with primary antibodies against NF-κB p65 or Vimentin diluted in 1% bovine serum. Following PBS washes, cells were incubated with Incubate goat anti-mouse IgG Dylight 488 (1:500) or anti-rabbit IgG Dylight 488 (1:500) in the dark (2 h), counterstained with DAPI (10 min), and imaged using an inverted fluorescence microscope (Olympus, Tokyo, Japan).

### 4.7. Western Blot

ARPE-19 cells were treated with GNP (200, 400, and 600 μg/mL, 24 h), followed by exposure to H_2_O_2_ (800 μM). After 24 h incubation, cells were washed with PBS and lysed using RIPA buffer supplemented with PMSF (100:1). Protein concentrations were determined by BCA assay. Subsequently, proteins were separated by SDS-PAGE and transferred onto nitrocellulose (NC) membranes. The membranes were blocked with 7% skim milk for 2 h at room temperature, then incubated overnight at 4 °C with primary antibodies (mouse anti-antibody at 1:500 dilution; rabbit anti-antibody at 1:1000 dilution). After washing, membranes were incubated with corresponding HRP-conjugated secondary antibodies (anti-mouse at 1:1000; anti-rabbit at 1:2000) at room temperature (2 h). Protein expression levels were finally visualized using an ECL detection system (Syngene, Cambridge, UK).

### 4.8. Statistical Analysis

GraphPad Prism 8.0 (GraphPad Software, San Diego, CA) and Image J (version 1.46R, NIH) were used for data analyses. All data were presented as mean ± standard deviation (SD) (*n* = 3–4) and repeated three times. Statistical analysis by one-way analysis of variance (ANOVA) was accompanied by Dunnett’s multiple comparison test for group comparison. The statistical significance of all tests was *p* < 0.05.

## 5. Conclusions

In conclusion, GNP inhibits H_2_O_2_-induced oxidative stress and EMT in ARPE-19 cells at the molecular level. Oxidative damage, inflammation, apoptosis, and EMT typically coexist and interact synergistically in pathological conditions. Our study reveals two key findings: First, GNP demonstrates significant antioxidant properties, functioning as an effective free radical scavenger. Mechanistically, it modulates the NF-κB signaling pathway by suppressing nuclear translocation of p65 and phosphorylation of IκB, while simultaneously downregulating pro-apoptotic Bax expression and upregulating anti-apoptotic Bcl-2 expression, thereby attenuating both inflammatory responses and apoptotic cell death. Second, GNP effectively inhibits cell migration, indicating its potential to mitigate EMT progression. These findings collectively indicate that GNP, a sulfated polysaccharide, possesses considerable antioxidant capacity. In spite of the promising protective effects of GNP demonstrated in this study, it is important to acknowledge its limitations, particularly regarding the depth of mechanistic investigation and the generalizability of the model. Future work will prioritize the use of canonical inducers (e.g., TGF-β), establish antioxidant benchmarks (e.g., NAC), and quantify caspase activity to precisely elucidate the molecular pathways. Further in-depth animal and pharmacological studies will be essential to consolidate the therapeutic potential of GNP for dry AMD. Furthermore, this study provides a scientific foundation for the value comprehensive utilization of *Gelidium crinale* in nutraceutical and pharmaceutical applications.

## Data Availability

Data will be made available on request.
